# The Complete Mitochondrial Genome of *Yarrowia Lipolytica*

**DOI:** 10.1002/cfg.72

**Published:** 2001-04

**Authors:** Stefan Kerscher, Gregor Durstewitz, Serge Casaregola, Claude Gaillardin, Ulrich Brandt

**Affiliations:** 1 Universitätsklinikum Frankfurt Institut für Biochemie I Zentrum der Biologischen Chemie Frankfurt am Main D-60590 Germany; 2 Laboratoire de Biologie Moléculaire et Cellulaire INRA-CNRS Institute National Agronomique Thiverval Grignon 78850 France; 3 Universitätsklinikum Frankfurt Institut für Biochemie I Zentrum der Biologischen Chemie Theodor-Stern-Kai 7, Haus 25 B Frankfurt am Main D-60590 Germany; 4 Technische Universität München Institut für Tierzucht und Molekulare Genetik Freising-Weihenstephan D-85350 Germany

## Abstract

We here report the complete nucleotide sequence of the 47.9 kb mitochondrial (mt) genome from the obligate aerobic yeast *Yarrowia lipolytica*. It encodes, all on the same strand, seven subunits of NADH: ubiquinone oxidoreductase (ND1-6, ND4L), apocytochrome *b* (COB), three subunits of cytochrome oxidase (COX1, 2, 3), three subunits of ATP synthetase (ATP6, 8 and 9), small and large ribosomal RNAs and an incomplete set of tRNAs. The *Y. lipolytica* mt genome is very similar to the *Hansenula wingei* mt genome, as judged from blocks of conserved gene order and from sequence homology. The extra DNA in the *Y. lipolytica* mt genome consists of 17 group 1 introns and stretches of A+Trich sequence, interspersed with potentially transposable GC clusters. The usual mould mt genetic code is used. Interestingly, there is no tRNA able to read CGN (arginine) codons. CGN codons could not be found in exonic open reading frames, whereas they do occur in intronic open reading frames. However, several of the intronic open reading frames have accumulated mutations and must be regarded as pseudogenes. We propose that this may have been triggered by the presence of untranslatable CGN codons. This sequence is available under EMBL Accession No. AJ307410.


**Comparative and Functional Genomics**
*Comp Funct Genom* 2001; **2**: 80–90.
**DOI**: 10.1002/cfg.72

**Research Article**

### The complete mitochondrial genome of *Yarrowia lipolytica*

Stefan Kerscher[1]*, Gregor Durstewitz[1,†], Serge Casaregola[2], Claude Gaillardin[2] and Ulrich Brandt[1]
[1] *Universitätsklinikum Frankfurt, Institut für Biochemie I, Zentrum der Biologischen Chemie, D-60590 Frankfurt am Main, Germany*
[2] *Laboratoire de Biologie Moléculaire et Cellulaire INRA-CNRS, Institute National Agronomique, 78850 Thiverval Grignon, France*

**Correspondence to:*
*S. Kerscher, Universitätsklinikum Frankfurt, Institut für Biochemie I, Zentrum der Biologischen Chemie, Theodor-Stern-Kai 7, Haus 25 B, D-60590 Frankfurt am Main, Germany.*
*E-mail: kerscher@zbc.klinik.uni-frankfurt.de*

*† Current address:*
*Technische Universität München, Institut für Tierzucht und Molekulare Genetik, D-85350 Freising-Weihenstephan, Germany.*

Received: 3 January 2001
Accepted: 10 February 2001

### Abstract

**We here report the complete nucleotide sequence of the 47.9 kb mitochondrial (mt) genome from the obligate aerobic yeast *Yarrowia lipolytica*. It encodes, all on the same strand, seven subunits of NADH : ubiquinone oxidoreductase (ND1-6, ND4L), apocytochrome *b* (COB), three subunits of cytochrome oxidase (COX1, 2, 3), three subunits of ATP synthetase (ATP6, 8 and 9), small and large ribosomal RNAs and an incomplete set of tRNAs. The *Y. lipolytica* mt genome is very similar to the *Hansenula wingei* mt genome, as judged from blocks of conserved gene order and from sequence homology. The extra DNA in the *Y. lipolytica* mt genome consists of 17 group 1 introns and stretches of A + T-rich sequence, interspersed with potentially transposable GC clusters. The usual mould mt genetic code is used. Interestingly, there is no tRNA able to read CGN (arginine) codons. CGN codons could not be found in exonic open reading frames, whereas they do occur in intronic open reading frames. However, several of the intronic open reading frames have accumulated mutations and must be regarded as pseudogenes. We propose that this may have been triggered by the presence of untranslatable CGN codons. This sequence is available under EMBL Accession No. AJ307410. Copyright © 2001 John Wiley & Sons, Ltd.**

**Keywords:** ***Yarrowia lipolytica*; mitochondrial genome; complex I; NADH : ubiquinone oxidoreductase; NADH dehydrogenase; tRNA; CGN codon; intronic ORF**

### Introduction

Mitochondrial genomes from different eukaryotic lineages show an astonishing degree of diversity in size, gene content and genome organization. In recent years, systematic sequencing of protist and fungal mitochondrial genomes, mainly through the efforts of the Organelle Megasequencing Program (Gray *et al.*, 1998) and the Fungal Mitochondrial Genome Project (Paquin *et al.*, 1997) has yielded valuable information on ancestral and derived mitochondrial (mt) genomes and their evolutionary relationships. While ancestral mt genomes, as found in the jakobid flagellate *Reclinomonas americana* (Lang *et al.*, 1997), contain a large number of additional genes encoding components of their own transcription/translation machinery, derived mt genomes, as found in animals and higher fungi, are characterized by a much reduced gene content, which typically consists of the genes encoding hydrophobic subunits of respiratory chain complexes, large and small ribosomal RNAs and a full or partial set of tRNAs (Gray *et al.*, 1999).

Among the mt genomes of ascomycetous fungi, there is some variability with respect to gene content. Seven genes encoding hydrophobic subunits of NADH : ubiquinone oxidoreductase (complex I) are present in most cases but absent in species that lack this enzyme, such as *Saccharomyces cerevisiae* (Foury, 1998) and *Schizosaccharomyces pombe* (Lang, 1993). The ATP9 gene is present in the mt genome of most species, as in *S. cerevisiae* (Foury, 1998), but not in *Podospora anserina* (Cummings *et al.*, 1990). Other fungi, such as *Neurospora crassa* and *Aspergillus nidulans*, have both a mitochondrial

Copyright © 2001 John Wiley & Sons, Ltd.

and a nuclear gene for ATP9, with the nuclear gene being the active one (van den Boogaart *et al*., 1982; Brown *et al*., 1985). Some fungal mt genomes also contain additional genes, encoding accessory ribosomal proteins such as VAR1 in *S. cerevisiae* (Hudspeth *et al*., 1982), *Torulopsis glabrata* (Ainley *et al*., 1985) and *H. wingei* (Okamoto *et al*., 1994; Sekito *et al*., 1995) or SP5 in *N. crassa* (Collins, 1993), or the RNA component of RNase P in *S. cerevisiae* (Foury, 1998). The mould mitochondrial genetic code appears to be well conserved, with the UGA codon being read as tryptophan as the only exception from the universal genetic code. Two notable exceptions are mitochondria from *S. cerevisiae*, where AUA is read as methionine and CUN is read as threonine, and from *Sz. pombe*, where, as in plant mitochondria, the universal genetic code is used (Dirheimer and Martin, 1990; Lang, 1993; Paquin *et al*., 1997).

*Yarrowia lipolytica* is an obligate aerobic, ascomycetous yeast which can efficiently be manipulated by classical and molecular genetic techniques. It can utilize a range of unusual hydrophobic carbon sources, including alkanes like *n*-hexadecane (Barth and Gaillardin, 1996, 1997). *Y. lipolytica*'s proficiency in secreting high amounts of an alkaline extracellular protease encoded by the XPR2 gene has been exploited for the production of heterologous proteins under the control of XPR2 hybrid promoters (Madzak *et al*., 1999, 2000).

In contrast to *S. cerevisiae*, which is adapted to alcoholic fermentation (Lagunas, 1986), mitochondrial respiration is essential for *Y. lipolytica*. Also in contrast to *S. cerevisiae*, proton-translocating NADH : ubiquinone oxidoreductase (complex I) is present in the respiratory chain of *Y. lipolytica* mitochondria (Kerscher *et al*., 1999). Owing to its genetic versatility and superior protein stability, *Y. lipolytica* has recently been established as a powerful new model system for the analysis of complex I (Ahlers *et al*., 2000; Djafarzadeh *et al*., 2000). In all eukaryotes containing complex I, seven of its hydrophobic subunits are mitochondrially encoded. Since so far only the sequence of a 6.6 kb *Spe*I–*Bgl*II fragment, containing the genes for ATP8, ATP6, COX3, ND4 and several tRNAs, has been sequenced (GenBank Accession No. L15359) and functionally characterized (Matsuoka *et al*., 1994a, 1994b), we set out to analyse the complete sequence of the mt genome from *Y. lipolytica*.

### Materials and methods

The *Y. lipolytica* mt DNA sequence was generated from two closely related strains, wild-type isolate W29 and laboratory strain E150 (MatB, *his*-1, *leu2*-270, *ura3*-302, *xpr2*-322). Owing to the fact that the predecessors of strain E150 had been made isogenic by several rounds of backcrossing to strains derived from W29 (Barth and Gaillardin, 1996), the mt DNA sequences of W29 and E150 were found to be virtually identical. There is up to 1% divergence in the non-coding part of introns and in intergenic regions, much less in exonic sequences. The sequence published here is the one found in strain W29. Shotgun sequencing of genomic DNA from wild-type strain W29 was as described in Artiguenave *et al*. (2000) and Tekaia *et al*. (2000). This resulted in six large contigs containing up to 200 sequence reads and covering almost 96% of the mt genome. Gaps were closed by polymerase chain reaction (PCR) sequencing of PCR products generated with W29 total DNA.

Mitochondrial DNA from strain E150 was isolated from total DNA by caesium chloride density gradient centrifugation in the presence of bisbenzimide (Hoechst 33258), as described in (Kraiczy *et al*., 1996). Individual *Hin*dIII fragments were subcloned into pBluescriptSK$^-$ (Stratagene) and sequenced using the BigDye™ Terminator Cycle Sequencing Ready Reaction Kit (PE Applied Biosystems) and analysed on an ABI 310 genetic analyser (PE Applied Biosystems); 88% of the *Y. lipolytica* mt genome was sequenced in this way.

Sequences were analysed using the DNASIS (Hitachi) and HUSAR (DKFZ, Heidelberg, Germany) programme packages. Nineteen of the 27 tRNAs (T, E, M1, M2, Q, K1, I1, S1, A, F, N, G, V, D, K2, W, L3, P, R) were identified using the programme FAStRNA (El-Mabrouk and Lisacek, 1996) at **http://bioweb.pasteur.fr.seqanal/interfaces/fastrna.html**.

For N-terminal sequencing of *Y. lipolytica* mt proteins, mt membranes were prepared as described (Kerscher *et al*., 1999) and subjected to preparative two-dimensional electrophoresis (blue native polyacrylamide gel electrophoresis, BN–PAGE; denaturing polyacrylamide gel electrophoresis, SDS–PAGE). Individual proteins were electroblotted onto Immobilon P membranes, incubated for 24 h at 37°C in a 1 : 1 mixture of trifluoroacetic acid and methanol for deformylation and sequenced directly using a

Copyright © 2001 John Wiley & Sons, Ltd.

473 protein sequencer (Applied Biosystems) as described (Arnold *et al.*, 1998).

### Results and discussion

### Composition

The mitochondrial genome of *Y. lipolytica* strain W29 consists of a circular molecule with a size of 47.9 kb. This is intermediate between the compact 20 kb and 27.7 kb mt genomes of *T. glabrata* (Clark-Walker, 1992) and *H. wingei* (Okamoto *et al.*, 1994; Sekito *et al.*, 1995) and the large 100.3 kb mt genome of *P. anserina* (Cummings *et al.*, 1990). All genes are encoded on the same strand, as commonly observed among ascomycetous fungi, with the notable exception of *S. cerevisiae*, where a single tRNA gene (*thr1*) resides on the opposite strand (Foury, 1998).

The coding strand has a purine content of 53.0%. A *Bgl*II site upstream of the rnl gene was chosen as the start position for sequence numbering (see Figure 1).

Of the *Y. lipolytica* mt genome, 26.1% code for 14 subunits of respiratory chain complexes (exonic ORFs only), 13.2% code for the large and small ribosomal RNAs and a total of 27 functional tRNAs. The G+C content is 22.7% for the whole mt genome and 25.8% for the exonic ORFs.

### Protein encoding genes

The *Y. lipolytica* mt genome has a typical gene content, since it holds the 14 hydrophobic subunits of respiratory chain complexes commonly encoded in the mt genome of ascomycetous fungi. ND1-6 and ND4L are subunits of NADH : ubiquinone oxidoreductase (complex I), COB is the apocytochrome of ubiquinone : cytochrome oxidoreductase (complex III), COX1–3 are subunits of cytochrome oxidase (complex IV), and ATP6, ATP8 and ATP9 are subunits of ATP synthetase (complex V). Additional genes found in the mt DNA of other fungi, such as genes encoding ribosomal proteins or the RNA component of RNAse P, could not be detected. The sequence coordinates of the protein encoding genes in the *Y. lipolytica* mt genome are summarized in Table 1. All proteins have homologous counterparts in the mt genome of other ascomycetous fungi. Identity and similarity scores are summarized in Table 2. It should be noted, however, that due to the possibility of horizontal transfer of mitochondrial DNA between species (Marinoni *et al.*, 1999) and due to the unusual mode of mitochondrial inheritance (Piškur, 1994), nuclear and mitochondrial genomes may not always share the same evolutionary histories.

Sequence alignments also strongly supported the notion that the usual mould mitochondrial genetic code is used in the *Y. lipolytica* mt genome. This was further confirmed by the N-terminal sequences of COB, COX1, COX2, COX3 and ATP8 (see Table 3), which showed translation of a CUU codon to leucine and translation of a AUA codon to isoleucine. COX2 was found to be proteolytically processed by removal of six amino acids from the N-terminus. Translation of COX1, COX3 and ATP8 start at a standard AUG codon. This is probably true for the other genes as well. In the case of ND6, ND1, ATP6, COX3, ND4, ATP9, ND4L, COB, ND2 and ND3, the initiator AUG is preceded by a short stretch rich in adenine. In terms of conserved gene order, the *Y. lipolytica* mt genome is most similar to the *H. wingei* mt genome. Five gene clusters, namely ND6/ND1, COX1/ATP8/ATP6, ND4/ATP9/COX2, ND4L/ND5 and COB/ND2/ND3, are conserved between these two organisms. Interestingly, the COX1/ATP8/ATP6 cluster is also present in the genus *Saccharomyces*, where it represents a transcription unit (Groth *et al.*, 2000). This cluster appears to represent an ancient organization, presumably being present already in the common progenitor of *Yarrowia* and *Saccharomyces*.

### Introns and intronic ORFs

Fungal mt introns fall into two groups. Group 1 introns are characterized by a common secondary structure, which is due to base pairings between conserved internal sequence elements and the unique mechanism of their splicing reaction, which involves the attack of a guanine nucleotide at the 5′ end of the intron. Many group 1 introns contain ORFs that may be contiguous with the upstream exon or may be free-standing. These encode maturase or maturase/endonuclease proteins which may assist in the splicing reaction or allow homing into cognate intronless alleles. Most group 1 endonucleases belong to one of four families, characterized by their possession of sequences homologous to the LAGLIDADG, GIY–YIG,

Copyright © 2001 John Wiley & Sons, Ltd. 

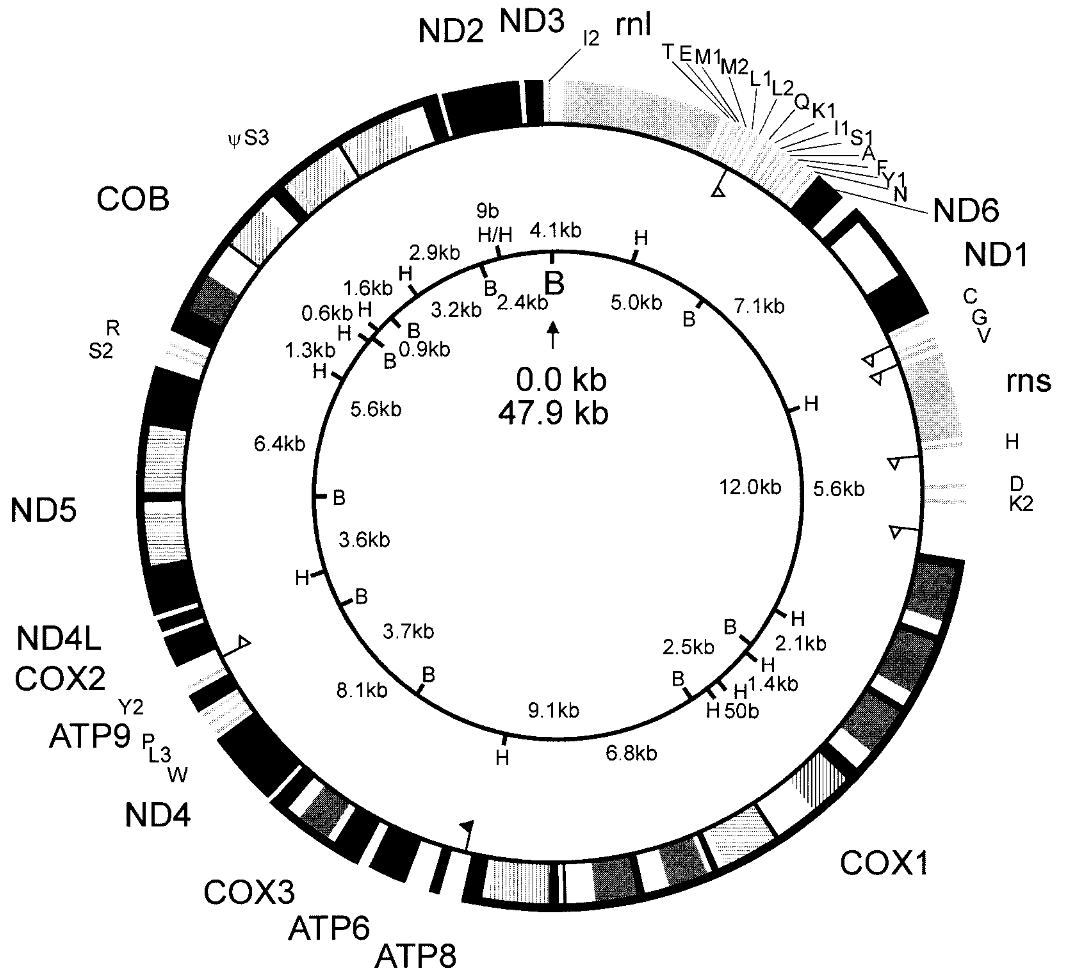

**Figure 1.** Map of the *Y. lipolytica* mt genome. Transcription is in clockwise direction. Exonic ORFs are shown in black, functional intronic ORFs in dark grey. Intronic ORFs that have become pseudogenes are hatched vertically; those that are untranslatable because they contain CGN codons are hatched horizontally. Large and small ribosomal RNAs (rnl, rns) and tRNAs (marked with their amino acid specificity in one-letter code) are shown in light grey. Restriction maps and the sizes of the corresponding fragments are shown for *Hin*dIII (H) and *Bgl*II (B). A *Bgl*II site upstream of the rnl gene defines base position one. Positions where the transcriptional start site motif ATATAAATA occurs at least once outside coding regions are marked with open flags, the transcriptional start identified by (Matsuoka *et al.*, 1994b) is marked with a filled flag

H–N–H or His–Cys box motifs. The mobility of group 2 introns depends on intron-encoded proteins with maturase, endonuclease and reverse transcriptase activity. For a review, see Belfort and Perlman (1995).

In the *Y. lipolytica* mt genome, the genes for ND1, COX1, COX3, ND5 and COB are interrupted by a total of 17 introns, all of which belong to group 1 (see Table 4). Splice junctions were deduced from the resulting exonic protein sequences. All conform to the rule that invariably the last base of the upstream exon is a pyrimidine and the last base of the intron is a guanine (Davies *et al.*, 1982), with the notable exception of the sixth intron in COX1. In this case, a different splice acceptor located 12 bp upstream of the proposed one might be used, which would result in the addition of the amino acid sequence YKIL between exons six and seven.

There is no intron in the rnl gene for the large ribosomal RNA that would correspond to the ω intron of *S. cerevisiae* mitochondria (Dujon, 1980). This had already been observed in Southern hybridization experiments (Jacquier and Dujon,

Copyright © 2001 John Wiley & Sons, Ltd.

Table 1. Location of protein-encoding genes in the mt genome of *Y. lipolytica*

| Gene | Start | End | Number of exons |
|---|---|---|---|
| ND6 | 5596 | 6153 | 1 |
| ND1 | 6408 | 8465 | 2 |
| COX1 | 13151 | 25609 | 10 |
| ATP8 | 26072 | 26218 | 1 |
| ATP6 | 26692 | 27459 | 1 |
| COX3 | 27760 | 29610 | 2 |
| ND4 | 29620 | 31080 | 1 |
| ATP9 | 31827 | 32057 | 1 |
| COX2 | 32582 | 33310 | 1 |
| ND4L | 33568 | 33837 | 1 |
| ND5 | 33838 | 38325 | 3 |
| COB | 39051 | 45791 | 5 |
| ND2 | 45833 | 47242 | 1 |
| ND3 | 47262 | 47648 | 1 |

1983). The single intron in ND1 shows extensive sequence similarity to the part of *P. anserina* ND1-I1 that lies downstream of a GIY–YIG endonuclease open reading frame (ORF), but does not contain a protein coding region. Also, there is no ORF in the short COX1-I8. The 15 remaining introns contain ORFs homologous to maturase or maturase/endonuclease proteins encoded in mt introns of other ascomycetous fungi (see Table 4).

Table 2. Sequence homology between mitochondrially encoded proteins from *Y. lipolytica* and other ascomycetous fungi (% similarity and identity)

| | *H. wingei* | | *P. anserina* | | *S. cerevisiae* | | *Sz. pombe* | |
|---|---|---|---|---|---|---|---|---|
| Protein | Sim. | Id. | Sim. | Id. | Sim. | Id. | Sim. | Id. |
| ND1 | 59 | 49 | 66 | 54 | – | – | – | – |
| ND2 | 52 | 34 | 54 | 38 | – | – | – | – |
| ND3 | 54 | 42 | 48 | 37 | – | – | – | – |
| ND4 | 56 | 44 | 56 | 43 | – | – | – | – |
| ND4L | 53 | 30 | 64 | 52 | – | – | – | – |
| ND5 | 54 | 44 | 62 | 49 | – | – | – | – |
| ND6 | 42 | 30 | 50 | 35 | – | – | – | – |
| COB | 75 | 66 | 72 | 61 | 76 | 65 | 69 | 58 |
| COX1 | 71 | 64 | 74 | 66 | 73 | 66 | 69 | 61 |
| COX2 | 75 | 66 | 74 | 59 | 79 | 67 | 63 | 53 |
| COX3 | 68 | 60 | 70 | 62 | 69 | 59 | 64 | 55 |
| ATP6 | 58 | 48 | 53 | 42 | 64 | 54 | 60 | 51 |
| ATP8 | 65 | 52 | 63 | 49 | 67 | 54 | 65 | 46 |
| ATP9 | 83 | 79 | – | – | 82 | 76 | 73 | 61 |

Values were created using the GAP programme at **http://genius.embnet.dkfz-heidelberg.de/** with a gap creation penalty of 8 and a gap extension penalty of 2.

Table 3. N-Terminal sequences of *Y. lipolytica* mt encoded proteins

| | |
|---|---|
| COB | maLRKKNs**L**LNmAN |
| COX1 | msLKLNIQ |
| COX2 | DVPVPYGLYF |
| COX3 | mNLtLKKFQ |
| ATP8 | mPQLvPFYFTNQ**I**F |

Shown in **bold** are a leucine in COB encoded by CUU and an isoleucine in ATP8 encoded by AUA. Lower case letters indicate amino acids that could not be identified unambiguously.

Interestingly, many of them have accumulated mutations that are either frameshifts, in-frame stop codons or small insertions and must be regarded as pseudogenes (see Figure 1). The functionality of two intronic ORFs that are not contiguous with the upstream exon (COX1-I6 and COX3-I1) is doubtful. It appears that the maturase function for the introns containing defective ORFs must be provided in *trans* by intron-encoded proteins that are still functional. Group 2 introns could not be found. Consequently, intron loss, which is believed to depend on the reverse transcriptase activity of group 2 intron encoded proteins (Belfort and Perlman, 1995), should not be feasible in the mt genome of the *Y. lipolytica* strains studied here.

### rRNAs and tRNAs

The genes for the large and small of ribosomal RNAs (*rnl*, nt position 72–3042 and *rns*, nt position 9333–10923) and their 5′ and 3′ boundaries were identified by their homology to their counterparts from *H. wingei*, *P. anserina* and other ascomycetous fungi.

Twenty-seven tRNA genes could be detected in the mt genome of *Y. lipolytica*. These are depicted in Figure 2. Interestingly, $tRNA^{Cys}$ from *Y. lipolytica* mitochondria, like $tRNA^{Cys}$ from *Sz. pombe* mitochondria (Dirheimer and Martin, 1990), can form a highly abnormal cloverleaf structure. A putative tRNA pseudogene, termed ψSer3 (see Figure 3), is found in the third COB intron, destroying the intronic ORF for a LAGLIDADG endonuclease.

The set of functional tRNAs in the *Y. lipolytica* mt genome is larger than the minimal set of 24 required for the translation of the mould mt genetic code, assuming that, as first proposed for *N. crassa* mitochondria, an unmodified U in the first anticodon position ('wobble position') can pair with all

Copyright © 2001 John Wiley & Sons, Ltd. 

Table 4. Introns and intronic ORFs in the *Y. lipolytica* mt genome

| Intron | 5′ Junction | 3′ Junction | | Intronic ORF | Related to |
|---|---|---|---|---|---|
| ND1-I1 | CTTTGTGGGT | tttaatgaag...gagattaacg | TACTATGGAC | NONE | *P. anserina* ND1-i1 |
| COX1-I1 | TCTTCTTCGT | caacaataga...tggtcatatg | CATGCCAGCT | GIY–YIG | (*P. anserina* COX1-i6) |
| COX1-I2 | AGGTTTTGGT | aaaataaggt...caataaaaag | AATTACTTAA | LAGLIDADG | *Sz. Cerevisiae* COX1-ai3 |
| COX1-I3 | GATGAACTGT | aaaaaaaata...aacaaattag | ATATTTCCCA | LAGLIDADG | *Sz. pombe* COX1-i1 |
| COX1-I4 | TTGATGAAAT | aaaataatat...aatattaatg | CACCCAGAGG | $\psi$ LAGLIDADG | *P. anserina* COX1-i8 |
| COX1-I5 | ACCCAGAGGT | aaaaataata...caaaataatg | ATATATTTTA | X LAGLIDADG | *S. pombe* COX1-i2 |
| COX1-I6 | TGTTTGAAGT | caagatggct...taaaatatta | CATCACATGT | F LAGLIDADG | *P. anserina* COX1-i12 |
| COX1-I7 | ATTCCACGAT | agaaaattaa...tagacttttg | TCATACTATG | LAGLIDADG | *P. anserina* COX1-i15 |
| COX1-I8 | TGTAGTAGCT | caaatgggcc...attattattg | CATTTCCACT | NONE | ??? |
| COX1-I9 | CTTCTTAGGT | caaatgtagg...gatgtaaaag | CTACAAGGAA | $\psi$ GIY–YIG | (*P. anserina* COX1-i14) |
| COX3-I1 | ATGTTCAGGT | gtttgcctga...cgaggatttg | GCCACATTAA | F LAGLIDADG | (*S. cerevisiae* COB-bi2) * |
| ND5-I1 | TATGGAGGGT | tggaataata...caacattttg | CCAACACCAG | X LAGLIDADG | *P. anserina* ND5-i2 |
| ND5-I2 | TACAATGAGT | caattggctc...tgttatttcg | CAACTTGGTA | X LAGLIDADG | *P. anserina* ND5-i3 |
| COB-I1 | TTTATGAGGT | aaatatatag...gttaaatttg | GCTACAGTTA | LAGLIDADG | *S. cerevisiae* COB-bi2 |
| COB-I2 | GAGGTGGGTT | taatttagag...caacaacatg | CTCAGTAGGT | $\psi$ LAGLIDADG | *S. cerevisiae* COB-bi3 |
| COB-I3 | TAAATTAGGT | caaggatggc...ataatttaag | CATCCAGATA | $\psi$ LAGLIDADG | (*S. cerevisiae* COX1-ai4) |
| COB-I4 | CAGCATCGAT | aaaagaagtc...aacaaatttg | AGTTCCAGAG | $\psi$ LAGLIDADG | (*S. cerevisiae* COX1-ai5) |

$\psi$, pseudogenes; X, ORF contains CGN codons; F, free-standing ORFs; *, described in Matsuoka *et al.* 1994a. Related introns were identified by BLAST searches of the NCBI mito databases at **http://www.ncbi.nlm.nih.gov/BLAST/** and by comparison of the flanking sequences. Introns with similar ORFs that are not inserted at corresponding positions in different organisms are bracketed.

four bases, while G can pair with U or C and a modified U can pair with A or G (Heckman *et al.*, 1980; Dirheimer and Martin, 1990). Apart from the two methionine tRNAs, the first of which by virtue of its homology to *S. cerevisiae* $tRNA^{f\text{-}Met}$ (Canaday *et al.*, 1980) seems to be specific for the initiation codon, there is only one additional pair (Leu2, Leu3) with identical anticodon sequences. Since $tRNA^{Leu2}$ lacks several hydrogen bonds in the D-, anticodon- and $\psi$-stems, its functionality is uncertain. Unlike the corresponding tRNA(UAG) from *S. cerevisiae*, where an insertion mutation has produced an unusual eight nucleotide anticodon loop and changed the aminoacylation specificity from leucine to threonine (Dirheimer and Martin, 1990), both $tRNA^{Leu2}$ and $tRNA^{Leu3}$ from *Y. lipolytica* possess the normal seven nucleotide anticodon loops. This is consistent with their function as leucine acceptors. In two cases, extra tRNAs are present, whose anticodons in the wobble positions differ from the expected sequence (Tyr2, Lys2). The case of $tRNA^{Tyr2}$ is particularly puzzling, since if as commonly found in fungal mt tRNAs (Dirheimer and Martin, 1990), the A in the first anticodon position was deaminated to I, this would result in a tRNA able to read through stop codons UAA and UAG. We conclude that *Y. lipolytica* mt $tRNA^{Tyr2}$, like the *S. cerevisiae* $tRNA^{Arg}$ specific for CGN codons, is one of the rare examples of an organellar tRNA containing an unmodified A in the first anticodon position.

Another remarkable species is $tRNA^{Ile2}$, which has an UAU anticodon. Although this tRNA is far from being perfectly base-paired, it appears to be functional. This finding is in contrast to the situation seen in *S. cerevisiae* mitochondria, where AUA is read as methionine by a tRNA(CAU) and in *Sz. pombe* mitochondria, where AUA is read as isoleucine by a tRNA(CAU) that probably contains a modified C in the first anticodon position allowing C:A wobble (Dirheimer and Martin, 1990). It should also be noted that the $tRNA^{Ile2}$ described here is different from the putative AUA-specific tRNA proposed to lie between the genes for ATP6 and ATP8 (Matsuoka *et al.*, 1994b). This sequence can indeed form a cloverleaf-like structure but, judged from the most unusual length and composition of its $\psi$-loop, it seems unlikely that it can function as a tRNA.

Most interestingly, the set of tRNAs in the *Y. lipolytica* mt genome appears to be incomplete. No $tRNA^{Arg}$ able to recognize CGN codons could be detected. We therefore analysed codon usage within the protein coding exons and the presumably

Copyright © 2001 John Wiley & Sons, Ltd. 

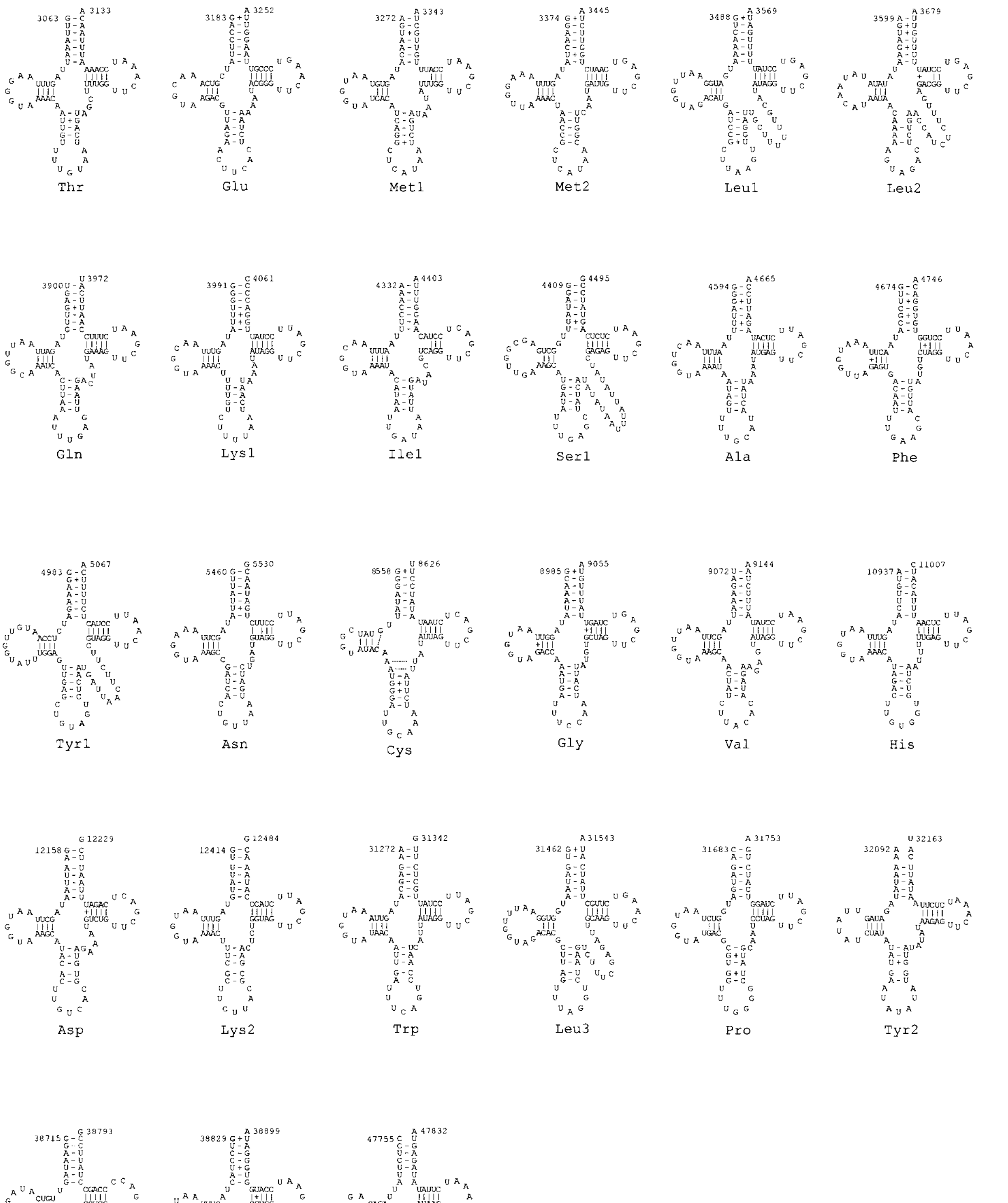

**Figure 2.** Cloverleaf structures of the 27 functional tRNA in the *Y. lipolytica* mt genome. G–C and A–T base pairings are depicted by bars, G–U pairings by cross signs

Copyright © 2001 John Wiley & Sons, Ltd.

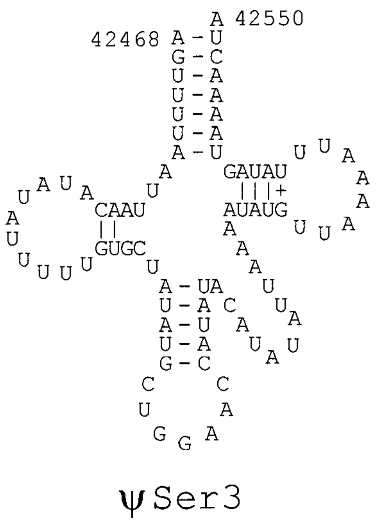

**Figure 3.** A hypothetical tRNA pseudogene (ψSer3) is inserted in COB-I3 of the *Y. lipolytica* mt genome functional intronic ORFs, i.e. those that are not pseudogenes and are contiguous with the upstream exon (see Table 5). Generally, codon usage in intronic ORFs is slightly less biased towards codons ending in A or U. The most striking observation, however, was that while CGN codons could not be found in exonic ORFs, they do occur in three intronic ORFs. CGC is found once in COX1-I5, while CGA is found once in ND5-I1 and three times in ND5-I2. We assume that the presence of 'forbidden' codons makes these intronic ORFs untranslatable, further reducing the number of functional intronic ORFs in the *Y. lipolytica* mt genome (see Figure 1).

Similarly, it has been reported that in the mt genome of *S. cerevisiae*, CGN codons are not found in the genes COB, COX1, COX2, COX3, ATP6 and ATP9 (Bonitz *et al.*, 1980). This is also true for ATP8, while in VAR1 (Hudspeth *et al.*, 1982), CGG and CGU are used once each. In intronic ORFs, arginine may be encoded as CGN, but such codons are much less frequent than AGR codons. A tRNA specific for CGN codons is present in the mt genome of *S. cerevisiae*, but appears to be a minor species compared to the tRNA that decodes AGR (Dirheimer and Martin, 1990). Notably, the anticodon of *S. cerevisiae* mt $tRNA^{Arg}$ is ACG, raising the question of how it interacts with CGA, CGG and CGC codons. Possibly, ORFs of the *S. cerevisiae* mt genome containing such codons can be translated with low efficiency only, if at all. This is reminiscent of the fact that in *Sz. pombe* mitochondria, out of a total of three intronic ORFs, two contain UGA codons that normally function as stop codons in this organism (Dirheimer and Martin, 1990; see also **http://megasun.bch.umontreal.ca/People/lang/FMGP/FMGP.html**).

The absence of a tRNA specific for CGN codons in the mt genome of *Y. lipolytica* could in theory be compensated by import of a nuclear coded tRNA

Table 5. Codon usage in exonic (E) and functional intronic (I) ORFs in the *Y lipolytica* mt genome and anticodons of the tRNAs that decode them

| AA | Codon | E | I | TRNA | AA | Codon | E | I | tRNA | AA | Codon | E | I | tRNA | AA | Codon | E | I | TRNA |
|---|---|---|---|---|---|---|---|---|---|---|---|---|---|---|---|---|---|---|---|
| **F** | UUU | 160 | 102 | | **S** | UCU | 78 | 49 | | **Y** | UAU | 192 | 175 | AUA | **C** | UGU | 30 | 26 | |
| | UUC | 160 | 15 | GAA | | UCC | 1 | 4 | | | UAC | 40 | 12 | GUA | | UGC | 0 | 5 | GCA |
| **L** | UUA | 611 | 231 | UAA | | UCA | 149 | 61 | UGA | **ter** | UAA | 12 | 7 | – | **W** | UGA | 57 | 31 | UCA |
| | UUG | 7 | 8 | | | UCG | 3 | 2 | | | UAG | 2 | 1 | – | | UGG | 2 | 5 | |
| **L** | CUU | 19 | 19 | | **P** | CCU | 65 | 31 | | **H** | CAU | 58 | 42 | | **R** | CGU | 0 | 0 | – |
| | CUC | 0 | 4 | | | CCC | 1 | 4 | | | CAC | 24 | 6 | GUG | | CGC | 0 | 1 | – |
| | CUA | 24 | 15 | UAG | | CCA | 68 | 17 | UGG | **Q** | CAA | 67 | 53 | UUG | | CGA | 0 | 4 | – |
| | CUG | 1 | 0 | | | CCG | 1 | 1 | | | CAG | 0 | 3 | | | CGG | 0 | 0 | – |
| **I** | AUU | 174 | 98 | | **T** | ACU | 105 | 73 | | **N** | AAU | 187 | 299 | | **S** | AGU | 98 | 49 | |
| | AUC | 87 | 17 | GAU | | ACC | 0 | 6 | | | AAC | 46 | 25 | GUU | | AGC | 4 | 2 | GCU |
| | AUA | 277 | 222 | UAU | | ACA | 128 | 42 | UGU | **K** | AAA | 80 | 271 | UUU | **R** | AGA | 75 | 73 | UCU |
| **M** | AUG | 118 | 37 | CAU | | ACG | 0 | 1 | | | AAG | 5 | 17 | CUU | | AGG | 0 | 8 | |
| **V** | GUU | 89 | 32 | | **A** | GCU | 162 | 36 | | **D** | GAU | 83 | 111 | | **G** | GGU | 168 | 80 | |
| | GUC | 4 | 3 | | | GCC | 10 | 5 | | | GAC | 9 | 5 | GUC | | GGC | 0 | 5 | |
| | GUA | 160 | 45 | UAC | | GCA | 84 | 22 | UGC | **E** | GAA | 61 | 88 | UUC | | GGA | 83 | 34 | UCC |
| | GUG | 10 | 2 | | | GCG | 3 | 1 | | | GAG | 29 | 13 | | | GGG | 4 | 5 | |

Copyright © 2001 John Wiley & Sons, Ltd. 

into mitochondria, as has been reported for a variety of lower fungi and also the basidiomycete *Schizophyllum commune* (Paquin *et al.*, 1997), or by post-transcriptional editing of tRNAs, which has been demonstrated to occur in plants (Weber *et al.*, 1990) and in marsupials (Börner *et al.*, 1996). However, since CGN codons only occur in intronic ORFs in the *Y. lipolytica* mt genome, there is no need to invoke such hypothetical mechanisms. Rather, we propose that the loss of a tRNA able to read CGN codons initially rendered many of the intronic ORFs in the *Y. lipolytica* mt genome non-functional and that some of these subsequently were turned into pseudogenes.

### Intergenic regions, genome instability

Most of the DNA outside exonic and intronic ORFs consists of A+T rich sequences of low complexity. For example, in the single intron in ND1, six direct repeats of the tetranucleotide sequence AATT (starting at position 7064) are followed by ten direct repeats of the pentanucleotide sequence TATGT. There is evidence for plasticity of the latter repeat from comparison with strain E150, where it is found nine times only.

The A+T-rich intergenic regions of the *Y. lipolytica* mt genome are interspersed with GC clusters, many of which are arranged as inverted repeats (see Table 6). Similar to those found in the mt genome of *S. cerevisiae* (Foury, 1998) and other fungi, these may function as mobile genetic elements. Two 43 bp long, almost perfectly palindromic GC-rich clusters with identical sequences are found in the second intron of the COB gene, where they destroy the ORF for the intron-encoded maturase. Interestingly, both of these putative minitransposon sequences are flanked by adenine pairs, which may indicate that small direct repeats are generated at the target site.

Rearrangements involving tRNA genes may also represent a source of genome instability. This is evidenced by a direct repeat involving the last 23 bp of $tRNA^{Tyr1}$ and 62 bp of downstream DNA (positions 5045–5129). After a 187 bp long spacer, this sequence is repeated with only one mismatch within 85 bp.

### Future perspectives

Little is known about the sequence motifs that are involved in replication and expression of the *Y. lipolytica* mt genome. It has been shown by primer extension analysis (Matsuoka *et al.*, 1994b) that transcription of a polycistronic mRNA containing the genes for ATP8, ATP6, COX3 and ND4 starts at the nonanucleotide motif ATATAAATA,

Table 6. GC-rich clusters in the mt genome of *Y. lipolytica*

| Position | Sequence |
|---|---|
| 5–36 | CCCCCCTACATATAGTAGGGGGGGGGGGG |
| 4858–4893 | GCCTCCCTACCCCCTACTAGTAGGGGGTAGGGAGGC |
| 6191–6229 | GTCCCCCCCTACCCCCCCCCTACTAGTAGGGGGTAGGAC |
| 12263–12272 | CCCCCCCCCC |
| 12300–12307 | GGGGGGGG |
| 12351–12362 | CTGGCCCCCTCC |
| 12963–12974 | CCCCCCCGGCCC |
| 12992–13000 | GGGGGGGGG |
| 13074–13082 | GGGGGGGGG |
| 25849–25863 | CCCCCCCCCTCTCTC |
| 26473–26502 | GTCTATCTCTCCTCCTGACTCCCCCCCCCC |
| 33348–33363 | CGCCCCCCCTCCCCCC |
| 33411–33422 | GGGGGGGGGGGG |
| 35641–35673 | CCCCCCCCCCATTCATTTTTAATGGGGGGGGGGG |
| 38556–38566 | CCCATCCCCCC |
| 38600–38609 | CCCCCTTCCC |
| 41260–41303 | GCCTTCCCCCCCCCTCCTGTCATAAGACAGGAGGGGGGAGAAGGC |
| 41824–41867 | GCCTTCCCCCCCCCTCCTGTCATAAGACAGGAGGGGGGAGAAGGC |

Inverted repeats are marked with arrows.

Copyright © 2001 John Wiley & Sons, Ltd.

which is similar to (A/T)TATAAG(T/A)(A/T), the transcriptional start site in *S. cerevisiae* mitochondria (Edwards, Levens and Rabinovitz, 1983). A monocistronic RNA encoding ATP9 is generated by processing of the same primary transcript four nucleotides downstream to the 3′ end of tRNA(CCN) (Matsuoka *et al.*, 1994b) by the action of RNAseP (Morales *et al.*, 1989). These data contrast with the finding that the COX1/ATP8/ATP6 cluster represents a transcription unit in the mt genome of the genus *Saccharomyces* (Groth *et al.*, 2000).

The nonanucleotide motif ATATAAATA occurs singly or as part of small clusters many times in the *Y. lipolytica* mt genome, usually outside coding regions, but also forms six overlapping repeats in the 3′ part of the first intron of COX1. Positions where the ATATAAATA motif is found at least once outside coding regions are marked with flag symbols in Figure 1. It remains to be determined whether these sequences function as transcriptional start sites.

Many more questions are still open. For example, we were so far unable to identify origins of replication in the *Y. lipolytica* mt genome from sequence comparisons with the corresponding consensus patterns from *S. cerevisiae* (Baldacci *et al.*, 1984). However, the complete sequence of the *Y. lipolytica* mt genome as present in widely used laboratory strains should provide useful information for further studies on its function in the future.

### Acknowledgements

We are indebted to Roghieh Djafarzadeh-Andabili and Hermann Schägger for their help with the N-terminal sequencing of *Y. lipolytica* mitochondrial proteins. We would also like to thank G. Beyer and A. Böttcher for excellent technical assistance and N. Kashani-Poor for critically reading the manuscript.

### References

Ahlers P, Zwicker K, Kerscher S, Brandt U. 2000. Function of conserved acidic residues in the PSST-homologue of complex I (NADH : ubiquinone oxidoreductase) from *Yarrowia lipolytica*. *J Biol Chem* **275**: 23577–23582.

Ainley WM, Macreadie IG, Butow RA. 1985. *Var1* gene on the mitochondrial genome in *Torulopis glabrata*. *J Mol Biol* **184**: 565–576.

Arnold I, Pfeiffer K, Neupert W, Stuart RA, Schägger H. 1998. Yeast $F_1F_0$–ATP synthase exists as a dimer: identification of three dimer specific subunits. *EMBO J* **17**: 7170–7178.

Artiguenave F, Wincker P, Brottier P, *et al.* 2000. Genomic exploration of the ascomycetous yeasts. 2 Data generation and processing for the random genome sequencing. *FEBS Lett* (in press).

Baldacci G, Chérif-Zahar B, Bernardi G. 1984. The initiation of DNA replication in the mitochondrial genome of yeast. *EMBO J* **3**: 2115–2120.

Barth G, Gaillardin C. 1996. *Yarrowia lipolytica*. In *Non-conventional Yeasts in Biotechnology*, Wolf K (ed.). Springer: Berlin–Heidelberg; 313–388.

Barth G, Gaillardin C. 1997. Physiology and genetics of the dimorphic fungus *Yarrowia lipolytica*. *FEMS Microbiol Rev* **19**: 219–237.

Belfort M, Perlman PS. 1995. Mechanisms of intron mobility. *J Biol Chem* **270**: 30237–30240.

Bonitz SG, Coruzzi G, Thalenfeld BE, Tzagoloff A, Macino G. 1980. Assembly of the mitochondrial membrane system. Structure and nucleotide sequence of the gene coding for subunit 1 of yeast cytochrome oxidase. *J Biol Chem* **255**: 11927–11941.

Börner GV, Mörl M, Janke A, Pääbo S. 1996. RNA editing changes the identity of a mitochondrial tRNA in marsupials. *EMBO J* **15**: 5949–59457.

Brown TA, Waring RB, Scazzochio C, Davies RW. 1985. The *Aspergillus nidulans* mitochondrial genome. *Curr Genet* **9**: 113–117.

Canaday J, Dirheimer G, Martin RP. 1980. Yeast mitochondrial methionine initiator tRNA: characterization and nucleotide sequence. *Nucleic Acids Res* **8**: 1445–1457.

Clark-Walker GD. 1992. Evolution of mitochondrial genomes in fungi. *Int Rev Cytol* **141**: 89–127.

Collins RA. 1993. *Neurospora crassa* mitochondrial genes. In *Genetic Maps: Locus Maps of Complex Genomes*, O'Brien SJ (ed). Cold Spring Harbor Laboratory Press: Cold Spring Harbor; 3.33–3.35.

Cummings DJ, McNally KL, Domenico J, Matsuura ET. 1990. The complete sequence of the mitochondrial genome of *Podospora anserina*. *Curr Genet* **17**: 375–402.

Davies RW, Waring RB, Ray JA, Brown TA, Scazzochio C. 1982. Making ends meet: a model for RNA splicing in fungal mitochondria. *Nature* **300**: 719–724.

Dirheimer G, Martin RP. 1990. Mitochondrial tRNAs; Structure, modified nucleosides and codon reading patterns. In *Chromatography and Modifications of Nucleosides, Part B; Biological Roles and Function of Modification*, Gehrke CK, Kuo CK (eds). Elsevier: Amsterdam; B197–B264.

Djafarzadeh R, Kerscher S, Zwicker K, *et al.* 2000. Biophysical and structural characterization of proton-translocating NADH-dehydrogenase (complex I) from the strictly aerobic yeast *Yarrowia lipolytica*. *Biochim Biophys Acta* **1459**: 230–238.

Dujon B. 1980. Sequence of the intron and flanking exons of the mitochondrial 21S rRNA gene of yeast strains having different alleles at the *ω* and *rib*-1 loci. *Cell* **20**: 185–197.

Edwards JC, Levens D, Rabinovitz M. 1983. Analysis of transcriptional initiation of yeast mitochondrial DNA in a homologous *in vitro* transcription system. *Cell* **31**: 337–346.

El-Mabrouk N, Lisacek F. 1996. Very fast identification of RNA motifs in genomic DNA. Application to tRNA search in the yeast genome. *J Mol Biol* **264**: 46–55.

Foury F. 1998. The complete sequence of the mitochondrial genome of *Saccharomyces cerevisiae*. *FEBS Lett* **440**: 325–331.

Copyright © 2001 John Wiley & Sons, Ltd. 

Gray MW, Burger G, Lang BF. 1999. Mitochondrial evolution. *Science* **283**: 1476–1481.

Gray MW, Lang BF, Cedergren R, *et al.* 1998. Genome structure and gene content in protist mitochondrial DNAs. *Nucleic Acids Res* **26**: 865–878.

Groth C, Petersen RF, Piškur J. 2000. Diversity in organization and the origin of gene order in the genus *Saccharomyces*. *Mol Biol Evol* **17**: 1833–1841.

Heckman JE, Sarnoff J, Alzner-DeWeerd B, Yin S, RajBhandary UL. 1980. Novel features in the genetic code and codon reading patterns in *Neurospora crassa* mitochondria based on sequences of six mitochondrial tRNAs. *Proc Natl Acad Sci U S A* **77**: 3167–3170.

Hudspeth MES, Ainley WM, Shumard DS, Butow RA, Grossman LI. 1982. Location and structure of the *var1* gene on yeast mitochondrial DNA: nucleotide sequence of the *40.0* allele. *Cell* **30**: 617–626.

Kerscher S, Okun JG, Brandt U. 1999. A single external enzyme confers alternative NADH : ubiquinone oxidoreductase activity in *Yarrowia lipolytica*. *J Cell Sci* **112**: 2347–2354.

Kraiczy P, Haase U, Gencic S, *et al.* 1996. The molecular basis for the natural resistance of the cytochrome $bc_1$ complex from strobilurin-producing basidiomycetes to center $Q_P$ inhibitors. *Eur J Biochem* **235**: 54–63.

Lagunas R. 1986. Misconceptions about the energy metabolism of *Saccharomyces cerevisiae*. *Yeast* **2**: 221–228.

Lang BF. 1993. The mitochondrial genome of *Schizosaccharomyces pombe*. In *Genetic Maps: Locus Maps of Complex Genomes*, O'Brien SJ (ed). Cold Spring Harbor Laboratory Press: Cold Spring Harbor; 3.133–3.135.

Lang BF, Burger G, O'Kelly CJ, *et al.* 1997. An ancestral mitochondrial DNA resembling a eubacterial genome in miniature. *Nature* **387**: 493–497.

Madzak C, Blanchin-Roland S, Otero RC, Gaillardin C. 1999. Functional analysis of upstream regulating regions from the *Yarrowia lipolytica* XPR2 promoter. *Microbiology* **145**: 75–87.

Madzak C, Treton B, Blanchin-Roland S. 2000. Strong hybrid promoters and integrative expression/secretion vectors for quasi-constitutive expression of heterologous proteins in the yeast *Yarrowia lipolytica*. *J Mol Microbiol Biotechnol* **2**: 207–216.

Marinoni G, Manuel M, Petersen RF, Hvidtfeldt J, Sulo P, Piškur J. 1999. Horizontal Transfer of genetic material among Saccharomyces yeast. *J Bacteriol* **181**: 6488–6496.

Matsuoka M, Matsubara M, Kakehi M, Imanaka T. 1994a. Homologous maturase-like proteins are encoded within the group I introns in different mitochondrial genes specifying *Yarrowia lipolytica* cytochrome *c* oxidase subunit 3 and *Saccharomyces cerevisiae* apocytochrome *b*. *Curr Genet* **26**: 377–381.

Matsuoka M, Matsubara M, Inoue J, Kakehi M, Imanaka T. 1994b. Organization and transcription of the mitochondrial ATP synthase genes in the yeast *Yarrowia lipolytica*. *Curr Genet* **26**: 382–389.

Morales MJ, Wise CA, Hollingsworth MJ, Martin NC. 1989. Characterization of yeast mitochondrial RNase P: an intact RNA subunit is not essential for activity *in vitro*. *Nucleic Acids Res* **17**: 6865–6881.

Okamoto K, Sekito T, Yoshida K. 1994. The mitochondrial genome of yeast *Hansenula wingei* encodes NADH dehydrogenase subunit genes ND4L and ND5. *Mol Gen Genet* **243**: 473–476.

Piškur J. 1994. Inheritance of the yeast mitochondrial genome. *Plasmid* **31**: 229–241.

Paquin B, Laforest MJ, Forget L, *et al.* 1997. The fungal mitochondrial genome project: evolution of fungal mitochondrial genomes and their gene expression. *Curr Genet* **31**: 380–395.

Sekito T, Okamoto K, Kitano H, Yoshida K. 1995. The complete mitochondrial sequence of *Hansenula wingei* reveals new characteristics of yeast mitochondria. *Curr Genet* **28**: 39–53.

Tekaia F, Blandin G, Malpertuy A, *et al.* 2000. Genomic exploration of the ascomycetous yeasts. 3 Methods and strategies used for sequence analysis and annotation. *FEBS Lett* (in press).

van den Boogaart P, Samallo J, Agsteribbe E. 1982. Similar genes for a mitochondrial ATPase subunit in the nuclear and mitochondrial genomes of *Neurospora crassa*. *Nature* **298**: 187–189.

Weber F, Dietrich A, Weil J-H, Marechal-Drouard L. 1990. A potato mitochondrial isoleucine tRNA is coded for by a mitochondrial gene possessing a methionine anticodon. *Nucleic Acids Res* **18**: 5027–5030.

Copyright © 2001 John Wiley & Sons, Ltd.
